# Palladium-103 as a Theranostic Auger Electron Emitter: From Cyclotron Production to Preclinical SPECT Evaluation in Murine and Canine Models

**DOI:** 10.3390/ph19081140

**Published:** 2026-07-23

**Authors:** Aicha Nour Laouameria, Domokos Máthé, Anikó Kubovje, Péter Vajdovich, Gyula Balka, Mátyás Hunyadi, Ralf K. Bergmann, Christer Halldin, László Forgách, Ildikó Horváth, Krisztián Szigeti, Roland Psáder, Jan Rijn Zeevaart, Lóránt Csige, Zoltán Szűcs

**Affiliations:** 1 Doctoral School of Chemistry, University of Debrecen, Egyetem tér 1, 4032 Debrecen, Hungary; 2 HUN-REN Institute for Nuclear Research, Bem tér 18/c, 4026 Debrecen, Hungary; hunyadi@atomki.hu (M.H.); csige.lorant@atomki.hu (L.C.); zszucs@atomki.hu (Z.S.); 3 Department of Biophysics and Radiation Biology, HUN-REN TKI, Semmelweis University, 1094 Budapest, Hungary; mathe.domokos@semmelweis.hu (D.M.); kubovje.aniko@semmelweis.hu (A.K.); rkbergmann@web.de (R.K.B.); christer.halldin@ki.se (C.H.); forgach.laszlo@semmelweis.hu (L.F.); horvath.ildiko1@semmelweis.hu (I.H.); szigeti.krisztian@semmelweis.hu (K.S.); 4 In Vivo Imaging Advanced Core Facility, Hungarian Centre of Excellence for Molecular Medicine, 1094 Budapest, Hungary; 5 Department of Clinical Pathology and Oncology, University of Veterinary Medicine, 1078 Budapest, Hungary; vajdovich.peter@univet.hu; 6 Department of Pathology, University of Veterinary Medicine, 1078 Budapest, Hungary; balka.gyula@univet.hu; 7 Department of Clinical Neuroscience, Karolinska Institute, 171 77 Stockholm, Sweden; 8 Department of Internal Medicine, University of Veterinary Medicine, 1078 Budapest, Hungary; psader.roland@univet.hu; 9 NuMeRI, Nuclear Medicine Research Infrastructure NPC, Steve Biko Academic Hospital, Pretoria 0084, South Africa; 10 Applied Radiation, South African Nuclear Energy Corporation (Necsa), Pelindaba 0240, South Africa

**Keywords:** Palladium-103, Auger electron emitter, Theranostics, dry distillation, separation, NOTA, DOTA-TATE, SPECT/CT imaging

## Abstract

**Background/Objectives:** Palladium-103 (^103^Pd) is an Auger electron-emitting radionuclide with nanometer-scale penetration ranges that result in highly localized energy deposition, making it well suited for molecularly targeted radionuclide therapy. However, efficient production, separation, and in vivo evaluation workflows remain limited. This study aimed to establish a scalable workflow for the production, separation, purification, radiolabeling, and preclinical assessment of ^103^Pd for theranostic applications. **Methods:** ^103^Pd was produced via the ^103^Rh(p,n)^103^Pd reaction. An upgraded dry-distillation radionuclide separation equipment (RSE) enabled high-efficiency separation from irradiated rhodium foils, while a cotton-assisted acid recovery process yielded purified ^103^Pd suitable for radiolabeling. Chelation with NOTA and DOTA-TATE was performed, and radiochemical purity was assessed using iTLC, SPE, and a C18 column. In vivo SPECT/CT imaging was conducted in NMRI Nu/Nu mice and in a canine model with spontaneous liver metastatic spread of insulinoma, injected with [^103^Pd]Pd-labeled compounds. **Results:** The upgraded RSE achieved separation efficiencies of 64–86% and overall recovery yields of 81–94%, outperforming conventional wet-chemistry methods. Radiolabeling produced stable complexes with >95% radiochemical purity. SPECT/CT imaging confirmed in vivo stability in mice. In the canine model, a slight reduction in tumor size and increased glucose levels were observed during 24 days post-systemic application of 520 MBq of ^103^Pd-DOTA-TATE radioactivity. **Conclusions:** This study establishes a complete and efficient radiochemical and preclinical pipeline for ^103^Pd, demonstrating its feasibility as a theranostic radionuclide. The combination of high-yield production, robust separation chemistry, and localized Auger-mediated energy deposition highlights ^103^Pd as a promising candidate for future targeted radionuclide therapy applications.

## 1. Introduction

Radiopharmaceutical therapy aims to deliver radiation selectively to tumor cells while sparing healthy tissue, thereby minimizing off-target toxicity and enhancing therapeutic efficacy [1]. Conventional radionuclide therapies often employ beta (β)-emitters and alpha (α)-emitters (e.g., lutetium-177 (^177^Lu) or actinium-225 (^225^Ac)) [2,3], which release high-energy particles that deposit dose over millimeter ranges for beta particles and typically 40–100 μm for alpha particles, sufficient for treating bulky tumors but frequently causing collateral damage in surrounding healthy tissue [4]. Such limitations motivate the search for radionuclides capable of delivering radiation with subcellular precision to better target minimal residual disease, disseminated tumor cells, or micrometastases.

Auger electron-emitting radionuclides have emerged as promising candidates for such precision therapy [5]. Upon decay via electron capture or internal conversion, these nuclides release cascades of low-energy electrons (Auger electrons) with nanometer-scale penetration ranges, leading to extremely localized dose deposition, often within a few tens of nanometers [6,7]. This short-range deposition can induce lethal DNA damage when the radionuclide is placed in proximity to the cell nucleus, thereby minimizing exposure of neighboring healthy cells [8,9].

However, accumulating experimental and theoretical evidence indicates that the biological effectiveness of Auger electron emitters is not exclusively driven by nuclear DNA damage. Auger electron cascades can induce significant extranuclear effects through interactions with essential cellular structures, particularly mitochondrial membranes and protein–cofactor complexes of the mitochondrial electron transport chain. Disruption of these complexes can impair oxidative phosphorylation, induce mitochondrial dysfunction, and trigger downstream cell death pathways, including apoptosis, ferroptosis, and microtubule-associated apoptosis [10,11]. In addition, Auger electron-induced lipid peroxidation represents a potent mechanism of cytotoxicity, as tumor cells exhibit altered redox regulation and antioxidant defenses compared with normal tissues, rendering them more susceptible to oxidative membrane damage. Furthermore, low-energy electron-mediated processes may exert immunomodulatory or hormetic effects, enhancing tumor cell elimination by immune effector cells such as macrophages and cytotoxic T lymphocytes [12,13]. Collectively, these findings demonstrate that effective Auger electron therapy does not strictly require nuclear localization of the radionuclide and that clinically relevant cytotoxicity may arise from spatially distributed subcellular interactions.

Within this class, palladium-103 (^103^Pd) stands out due to its unique decay characteristics, combining low-energy photon emissions, which can be detected using dedicated high-sensitivity imaging systems despite their low abundance, with Auger electron emissions for cytotoxicity, thereby suggesting theranostic potential [14]. ^103^Pd (t_1/2_ = 16.99 days) decays by electron capture, releasing Auger electrons and X-rays (20–23 keV), to rhodium-103 metastable state (^103m^Rh) (t_1/2_ = 56.1 min), which undergoes an isomeric transition to stable rhodium-103 (^103^Rh) with the emission of additional Auger electrons and low-intensity gamma(γ)-rays [15,16]. The decay of both radionuclides is associated with substantial Auger electron emission. For ^103^Pd, the total Auger electron energy released is approximately 41.8 keV per decay, while associated photon emissions contribute approximately 16.3 keV per decay. Similarly, ^103m^Rh releases approximately 35.7 keV per decay as Auger electrons and 1.59 keV per decay as photon emissions. These characteristics result in highly localized energy deposition and support the therapeutic relevance of the ^103^Pd/^103m^Rh decay system for targeted Auger electron therapy. The principal photon emissions associated with the decay of ^103^Pd and ^103m^Rh are summarized in Table 1.

However, despite the theoretical advantages, translation of ^103^Pd into targeted radionuclide therapy has been limited, partly owing to challenges in its production and radiochemical processing.

The production of ^103^Pd from stable ^103^Rh using a cyclotron has been reported, mainly through the ^103^Rh(p,n)^103^Pd reaction [17,18]. Nonetheless, conventional wet-chemistry separation of ^103^Pd from rhodium targets is plagued by rhodium’s high chemical inertness, leading to low yields, long processing times, substantial radioactive waste, and difficulties scaling up [19], all of which hamper routine production for preclinical or clinical use.

To address these limitations, we developed an upgraded dry-distillation radionuclide separation equipment (RSE) that exploits the distinct vapor pressures of palladium and rhodium [20]. Together with a novel substrate-based recovery method, this approach enables efficient, scalable, and reproducible separation and purification of cyclotron-produced ^103^Pd, with the possibility of reusing target materials, a feature that improves cost-effectiveness and sustainability.

^103^Pd is already well-established in clinical practice in its metallic form as a brachytherapy source for localized prostate cancer irradiation [21]. Conceptually, this principle of localized brachytherapy can be extended to a microscale analog in the form of particulate radioembolization. Given the long-standing clinical use and favorable radiophysical characteristics of ^103^Pd, its application in a local radioembolic context, where radiation is confined to the microvasculature, represents a logical and translationally relevant extension of its current therapeutic use. Accordingly, we explored the feasibility of incorporating ^103^Pd into a particulate radiopharmaceutical system. Serum albumin microspheres, which are well-established and clinically validated for studies of the pulmonary circulation, provide a suitable carrier platform [22,23,24]. Following intravenous administration, HSA (human serum albumin) is preferentially trapped within the pulmonary capillary bed due to its size, resulting in highly localized deposition. This characteristic offers a potential therapeutic opportunity for the treatment of lung metastases or primary pulmonary malignancies, where microvessel embolization combined with localized Auger electron-mediated irradiation could yield effective tumor control.

**Figure 1:**
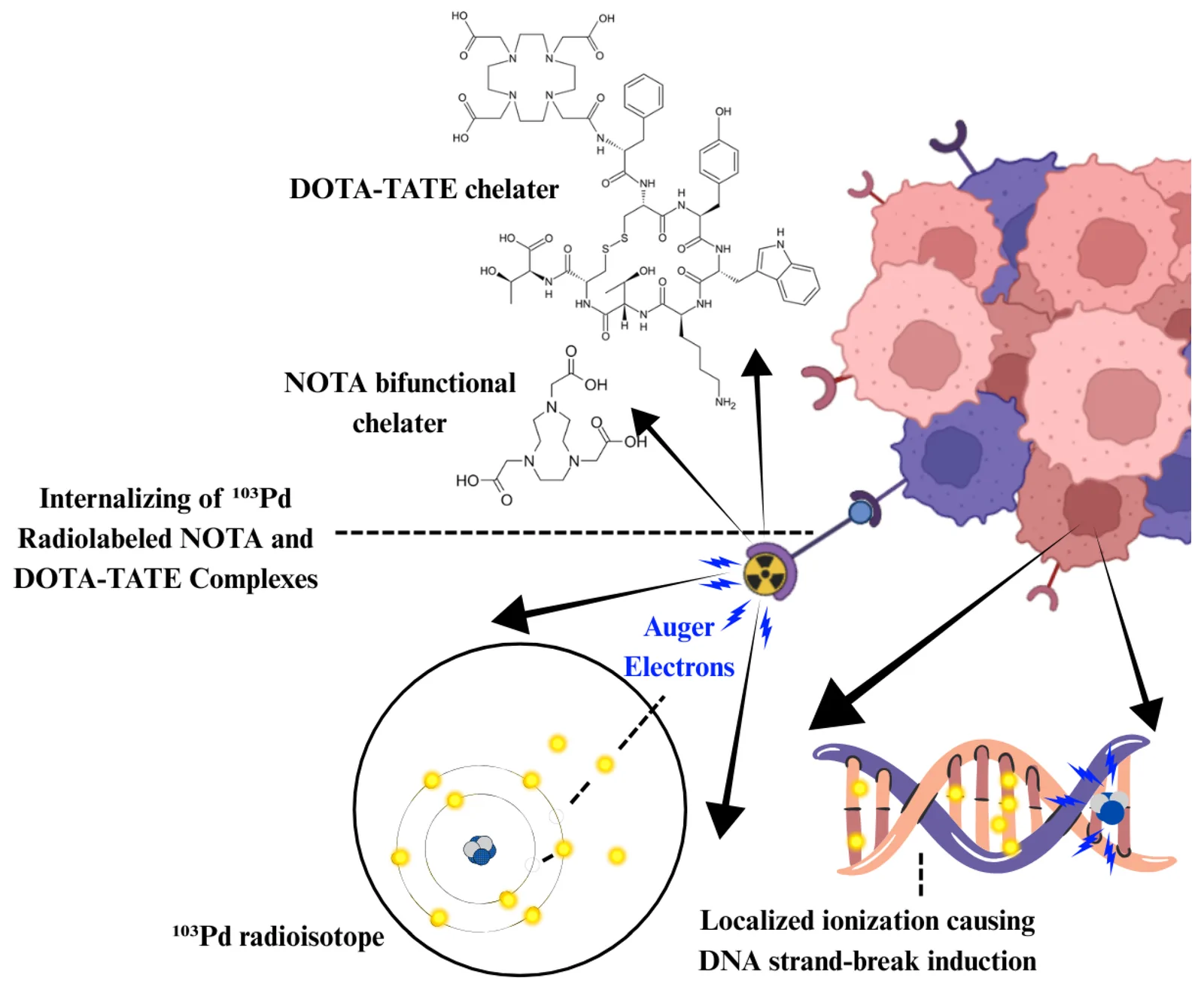
Mechanism of cellular delivery and Auger electron-induced cytotoxic effects of ^103^Pd-radiolabeled NOTA and DOTA-TATE complexes.

Moreover, while previous efforts have largely focused on brachytherapy applications or theoretical dosimetry, systematic in vivo evaluation of targeted, soluble, ^103^Pd radiolabeled molecular constructs also remains scarce. Preclinical imaging studies are essential to validate the chemical stability, pharmacokinetics, and targeting behavior of ^103^Pd conjugates. Accordingly, this study extends beyond radiochemistry: we evaluate [^103^Pd]Pd–NOTA complexes in murine models and [^103^Pd]Pd–DOTA-TATE complexes in a translational large-animal (canine) model with spontaneous insulinoma and metastatic liver tumors, to assess both imaging feasibility and preliminary therapeutic potential (Figure 1).

The bifunctional chelator 1,4,7-triazacyclononane-1,4,7-triacetic acid (NOTA) was employed for the complexation of ^103^Pd to all targeting biomolecules. Conjugation of NOTA to primary amine-containing biomolecules was performed using the widely adopted N-hydroxysuccinimide (NHS) ester activation method [25], ensuring efficient and site-selective attachment of the chelator.

DOTA-TATE is a well-characterized somatostatin receptor subtype-2 (SSTR2)-targeting peptide that has been widely used in peptide receptor radionuclide therapy (PRRT) when labeled with β-emitters or α-emitters. In this study, we selected DOTA-TATE as one of the chelator–biomolecule systems for ^103^Pd radiolabeling because it provides a clinically relevant targeting vector with high affinity for SSTR2, widespread tumor applicability (e.g., neuroendocrine tumors), and a robust development pipeline already established under other radionuclides (e.g., [^177^Lu]Lu– and [^225^Ac]Ac–DOTA-TATE) [26,27]. Although no previous studies have reported ^103^Pd-labeled DOTA-TATE, the proven chelation capacity of DOTA for radiometals, combined with the favorable chemical behavior of ^103^Pd, supports the rationale that [^103^Pd]Pd–DOTA-TATE conjugates could act as theranostic agents. Thus, applying ^103^Pd to both NOTA-conjugated biomolecules and DOTA-TATE not only maintains continuity with established chelation and targeting frameworks widely used in nuclear medicine, but also introduces the potential advantage of Auger-electron-based microdosimetry, a feature not previously explored for SSTR2-targeted or NOTA-functionalized systems.

This study aims to establish and validate a complete, scalable workflow for cyclotron production, dry-distillation separation, purification, radiolabeling, and in vivo preclinical evaluation of ^103^Pd, thereby demonstrating its feasibility as a theranostic Auger electron-emitting radionuclide for targeted cancer therapy and imaging.

## 2. Results

### 2.1. Optimized Production of ^103^Pd

**Figure 2:**
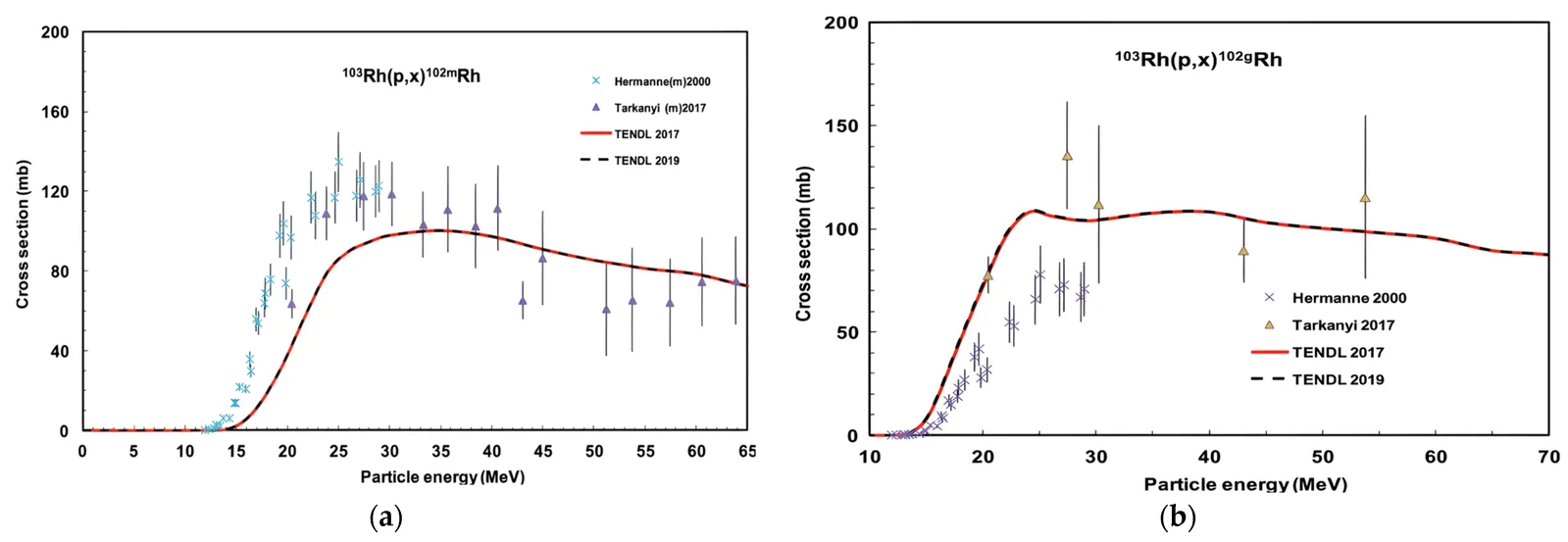
Experimental excitation-function data compared with TENDL-predicted cross-sections for: (**a**) the ^103^Rh(p,x)^102m^Rh reaction; (**b**) the ^103^Rh(p,x)^102g^Rh reaction. (Reproduced with permission from [28]).

After multiple irradiations, 11 MeV was identified as the optimal proton energy for ^103^Pd production. This energy lies near the peak of the ^103^Rh(p,n)^103^Pd excitation function and provides a favorable compromise between production yield and radionuclidic purity. Operation at 11 MeV achieves high cross-section for the desired ^103^Rh(p,n) channel while remaining below the thresholds of competing side reactions such as ^nat^Rh(p,pn)^102^/^102m^Rh and ^nat^Rh(p,p2n)^101^/^101m^Rh. These side nuclear reactions generate long-lived impurities (^101m^Rh, ^101^Rh, ^102m^Rh, ^102^Rh) that degrade the radionuclidic purity of the final product. The excitation-function data reported by Tárkányi et al. [28] (Figure 2), together with our experimental evaluations, clearly demonstrate that restricting the beam energy to approximately 11 MeV ensures efficient ^103^Pd generation while significantly suppressing formation of these undesirable radionuclidic by-products.

### 2.2. Separation Efficiency of ^103^Pd

The separation of ^103^Pd from irradiated ^103^Rh foils using the radionuclide separation equipment (RSE) resulted in yields ranging from 64% to 86% (Table 2). These values demonstrate the high efficiency of the diffusion-driven release and dry-distillation-based collection approach, representing a robust alternative to conventional wet-chemistry procedures. Traditional chemical dissolution and separation methods have been reported to suffer from lower overall ^103^Pd recovery and activity losses due to multi-step processing and incomplete chemical separation [19].

In contrast, the upgraded RSE, incorporating improved control of the heating and vacuum systems and an optimized copper-coated alumina ceramic crucible collection substrate, achieved significantly higher yields of ^103^Pd evaporation than the earlier RSE configuration [20]. This improvement of more than ~50% clearly confirms the superior performance of the upgraded system. Moreover, the enhanced RSE enables scalable processing while reducing reagent consumption, minimizing radioactive waste generation, and lowering overall operational complexity.

Moreover, after each dry-distillation cycle, the irradiated ^103^Rh foils were found to retain their mechanical integrity, mass, and surface morphology, indicating that the high-temperature and high-vacuum conditions did not induce measurable degradation. This structural stability enabled repeated reuse of the same foils for multiple irradiation and separation cycles, further improving process sustainability and cost-effectiveness.

### 2.3. Recovery Yield and Radionuclidic Purity

Recovery of ^103^Pd activity from the copper-coated alumina ceramic crucible substrate, following acidic extraction and subsequent purification, resulted in recovery efficiencies of 81–94%. These results confirm the effectiveness of this novel wiping-based recovery approach, which complements the RSE dry-distillation process and provides a fully integrated separation–recovery workflow. To our knowledge, this wiping-assisted acid extraction method has not been previously applied to dry-distilled palladium radionuclides, making it a novel and practical contribution to radiometal purification strategies. This combined strategy offers a promising alternative to conventional dissolution-based separation methods by enabling high recovery efficiencies with minimal substrate degradation and improved radionuclidic purity. The purified ^103^Pd solutions were subsequently transferred to Semmelweis University (Budapest, Hungary) for radiolabeling and in vivo studies.

### 2.4. Quality Control of ^103^Pd-Radiolabeled NOTA-Serum Albumin Microspheres Complexes

**Figure 3:**
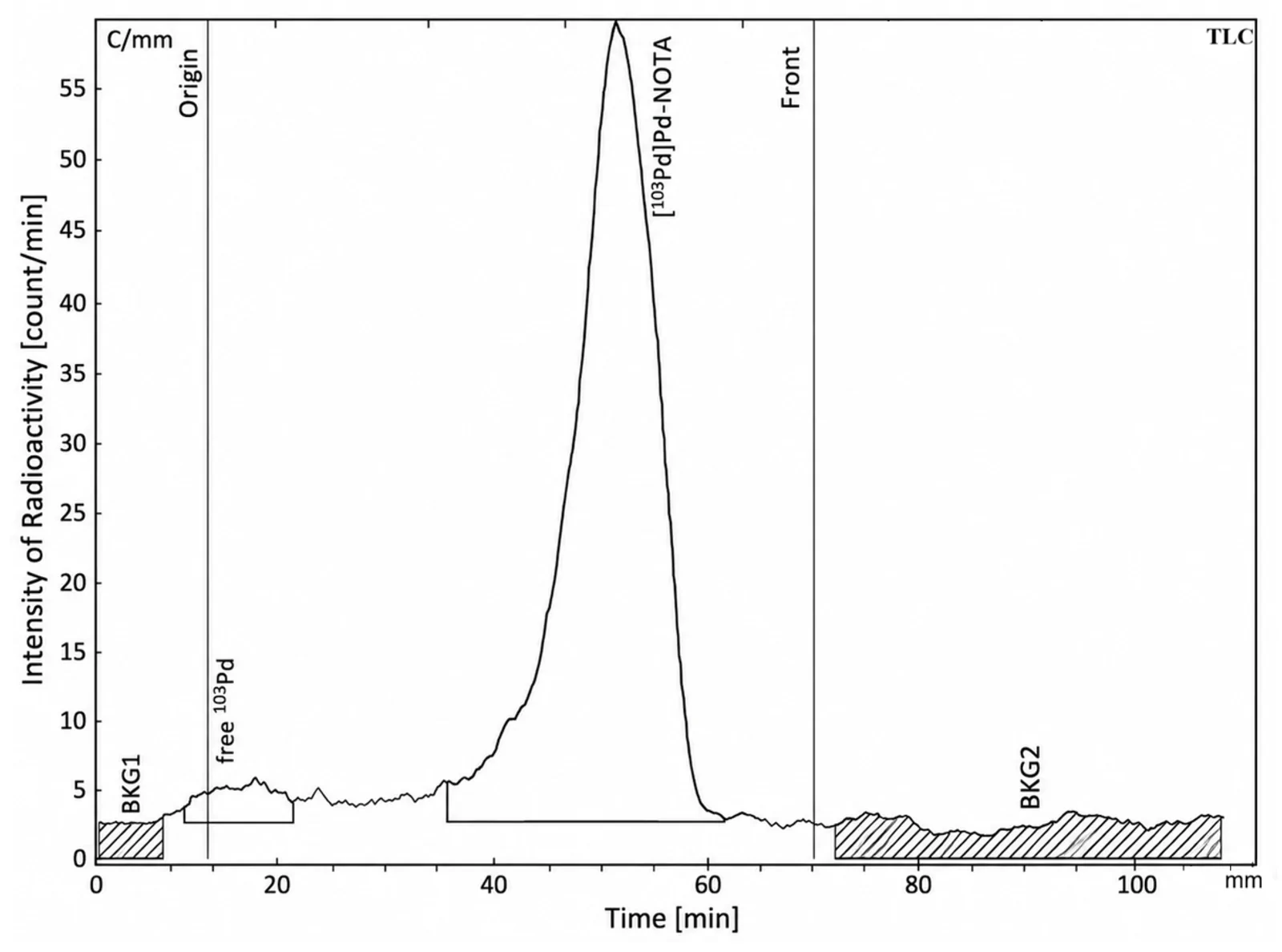
iTLC radiochromatographic analysis of [^103^Pd]Pd–NOTA.

Radiochemical yield (RCY) of the [^103^Pd]Pd–NOTA complexes was assessed by C18 mini-column chromatographic separation using methanol as the mobile phase, and the radiochemical purity (RCP) was assessed by instant thin-layer chromatography (iTLC) using ammonium acetate as the mobile phase. After labeling, the protein particles were centrifuged at 1200 *g* for 15 min, and the radioactivity of both pellet and supernatant was measured. The pellet was 4.674 MBq and the supernatant: 0.398 MBq, which confirms that the majority of the radioactivity was associated with the particles. RCP was 92.15% as determined by C18 mini-column chromatographic separation, and RCP was 95.7% as determined by iTLC. The signal corresponding to free palladium was negligible compared to the dominant [^103^Pd]Pd–NOTA complex signal (Figure 3), confirming successful radiolabeling and suitability of the [^103^Pd]Pd–NOTA complexes for injection into murine models.

### 2.5. Quality Control of ^103^Pd-Radiolabeled DOTA-TATE Complexes

Radiochemical purity (RCP) of the [^103^Pd]Pd–DOTA-TATE complex was assessed using two complementary methods: solid-phase extraction (SPE)-based affinity chromatography and instant thin-layer chromatography (iTLC).

For SPE analysis, a Sep-Pak mini affinity chromatography column was preconditioned with 20 mL of methanol followed by 20 mL of Milli-Q water. A drop of the reaction mixture was then loaded onto the column and sequentially eluted with 8 mL of methanol (peptide-bound fraction) and 10 mL of Milli-Q water (free or ionic palladium fraction). The radioactivity of each fraction was measured, and the RCP was calculated as the ratio of activity in the methanol fraction to the total activity recovered. Using this method, the RCP was determined to be approximately 92%.

In parallel, iTLC was performed using acetone as the mobile phase, in which the peptide-bound activity migrates with the solvent front. The chromatograms were analyzed using a Raytest Mini-GITA radio-TLC scanner (Raytest Isotopenmessgeräte GmbH, Straubenhardt, Germany). This method yielded an RCP of 96.38%, confirming efficient radiolabeling.

Following quality control, ~500 MBq of the [^103^Pd]Pd–DOTA-TATE solution was diluted with physiological saline to a final volume of 2 mL and administered intravenously for subsequent in vivo applications.

### 2.6. SPECT/CT Imaging of [^103^Pd]Pd–NOTA-HSA in Murines

**Figure 4:**
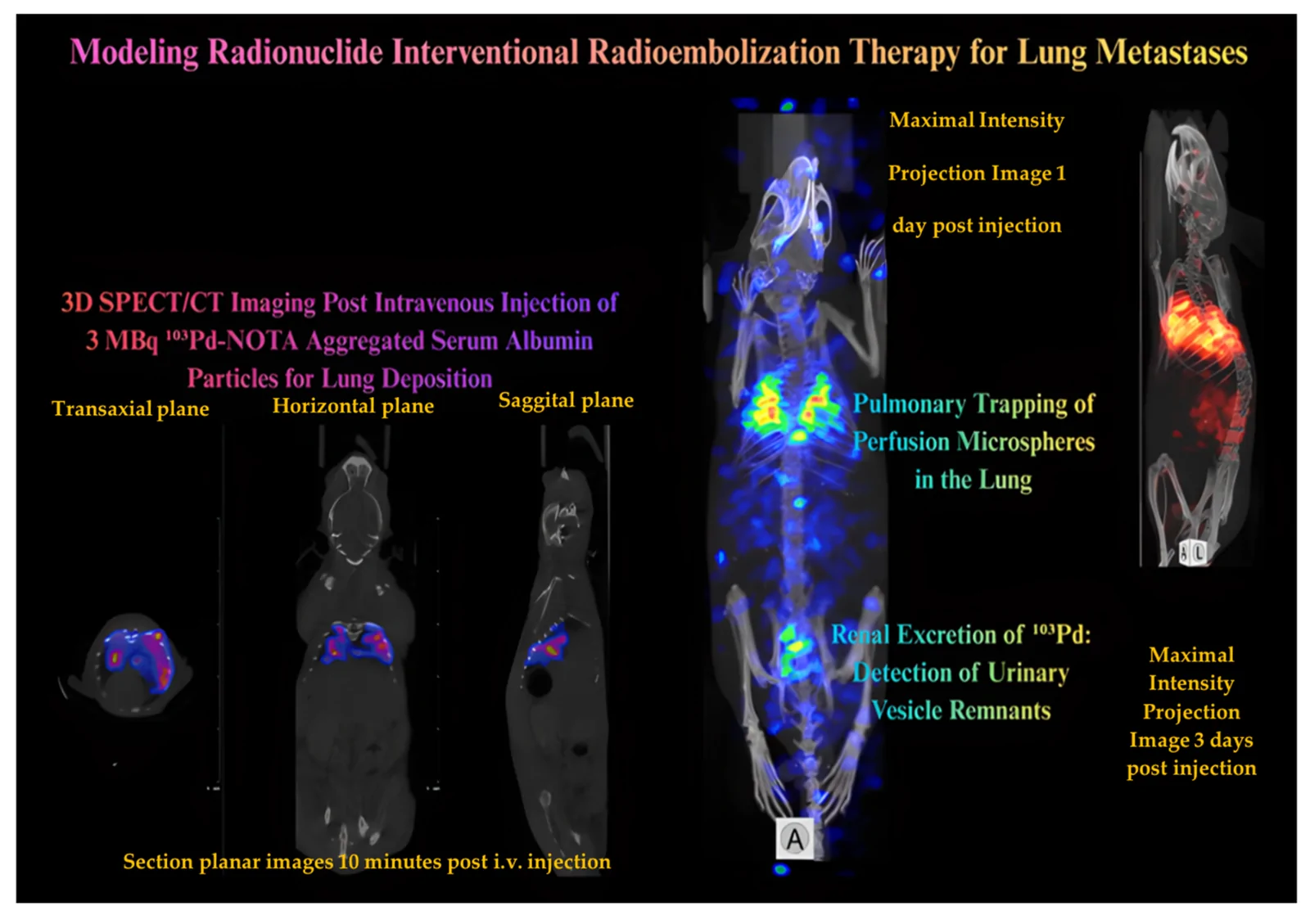
SPECT/CT images of [^103^Pd]Pd–NOTA-HSA particles in a murine model, illustrating in vivo accumulation in the lungs and excretion of decomposition products via the urinary system. The color scale represents SPECT activity, while grayscale corresponds to X-ray CT image reconstructions.

SPECT/CT imaging confirmed the expected ^103^Pd photon emissions, and semiquantitative tracer uptake was expressed as counts per milliliter (counts/mL). Serial acquisitions were performed at 10 min, 1 day, and 3 days post-injection. Figure 4 illustrates the organ-specific tracer accumulation at these post-injection time points. This investigation demonstrates the feasibility of using NOTA-functionalized serum albumin microspheres (prepared in-house using NOTA-NHS ester purchased from CheMatech, Dijon, France) radiolabeled with ^103^Pd for targeted delivery in particulate radioembolization of pulmonary metastases. In contrast to conventional microsphere therapies employing β-emitters such as yttrium-90 (^90^Y) or lutetium-177 (^177^Lu), which often result in broader radiation penetration and increased irradiation of healthy lung parenchyma [2,29], ^103^Pd provides a more confined dose distribution due to its low-energy emissions [14]. Furthermore, the short-range Auger electrons emitted by ^103^Pd enable highly localized intratumoral energy deposition, minimizing radiation exposure to adjacent normal tissues. These characteristics highlight the theranostic potential of ^103^Pd for targeted radiotherapy of lung lesions.

### 2.7. In Vivo SPECT/CT Evaluation of [^103^Pd]Pd–DOTA-TATE in a Canine Insulinoma with Spontaneous Metastatic Hepatic Malignancies

**Figure 5:**
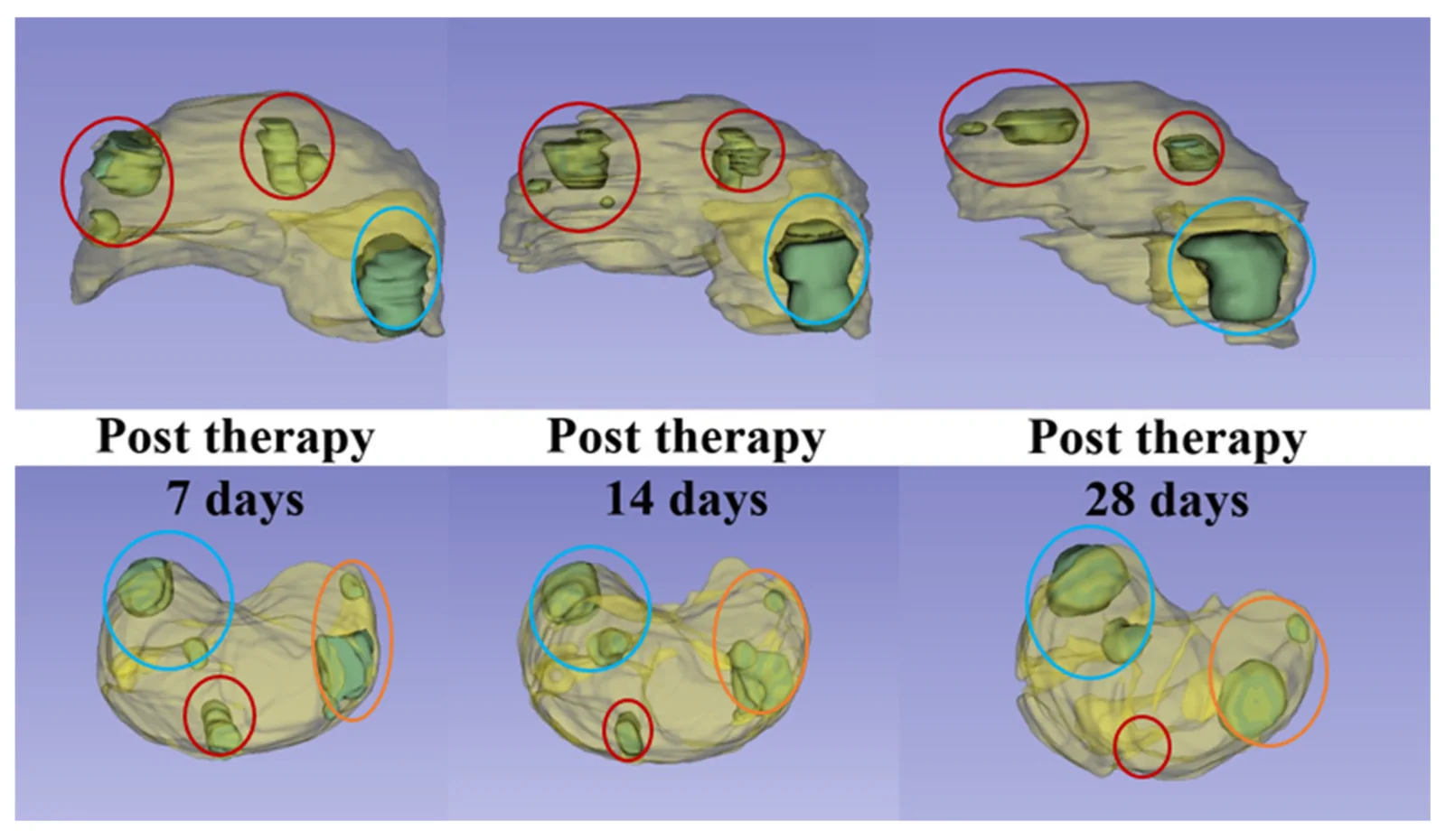
Threshold-based automated volumetric X-ray CT segmentation of metastatic liver tumor lesions in the dog at different time points post-therapy (7, 14, and 28 days). Red circles indicate tumor lesions that exhibited a reduction in size, blue circles indicate lesions that increased in size, and orange circles indicate lesions that remained stable without significant size change over the observation period.

Serial SPECT/CT scans performed at 1 h, 24 h, 1 week, 2 weeks, and 4 weeks after administration demonstrated a slight reduction in tumor size in general (Figure 5). Follow-up clinical evaluation showed a marked improvement in the animal’s previously hypoglycemic state due to the malignant insulinoma tumor producing supraphysiologic levels of glucose-lowering insulin. The dog had initially exhibited steroid therapy-resistant hypoglycemic bouts due to the original, histologically confirmed pancreatic insulin-secreting malignancy (insulinoma).

Consistent with the putative mechanism of effect of the targeted ^103^Pd therapy on insulin-secreting tumor cells, an increase in blood glucose levels was observed during the follow-up period. Continuous glucose monitoring (CGM) data showed an increase in average serum glucose levels from approximately 3.2 mmol·L^−1^ to 5.0 mmol·L^−1^ during the first two weeks of therapy, followed by stabilization at this level for a further two weeks. In contrast, insulin levels remained elevated after therapy. Clinically, the dog recovered strength and mobility, including the ability to walk using her hind limbs, representing a clear functional improvement compared to her debilitated state prior to treatment.

**Figure 6:**
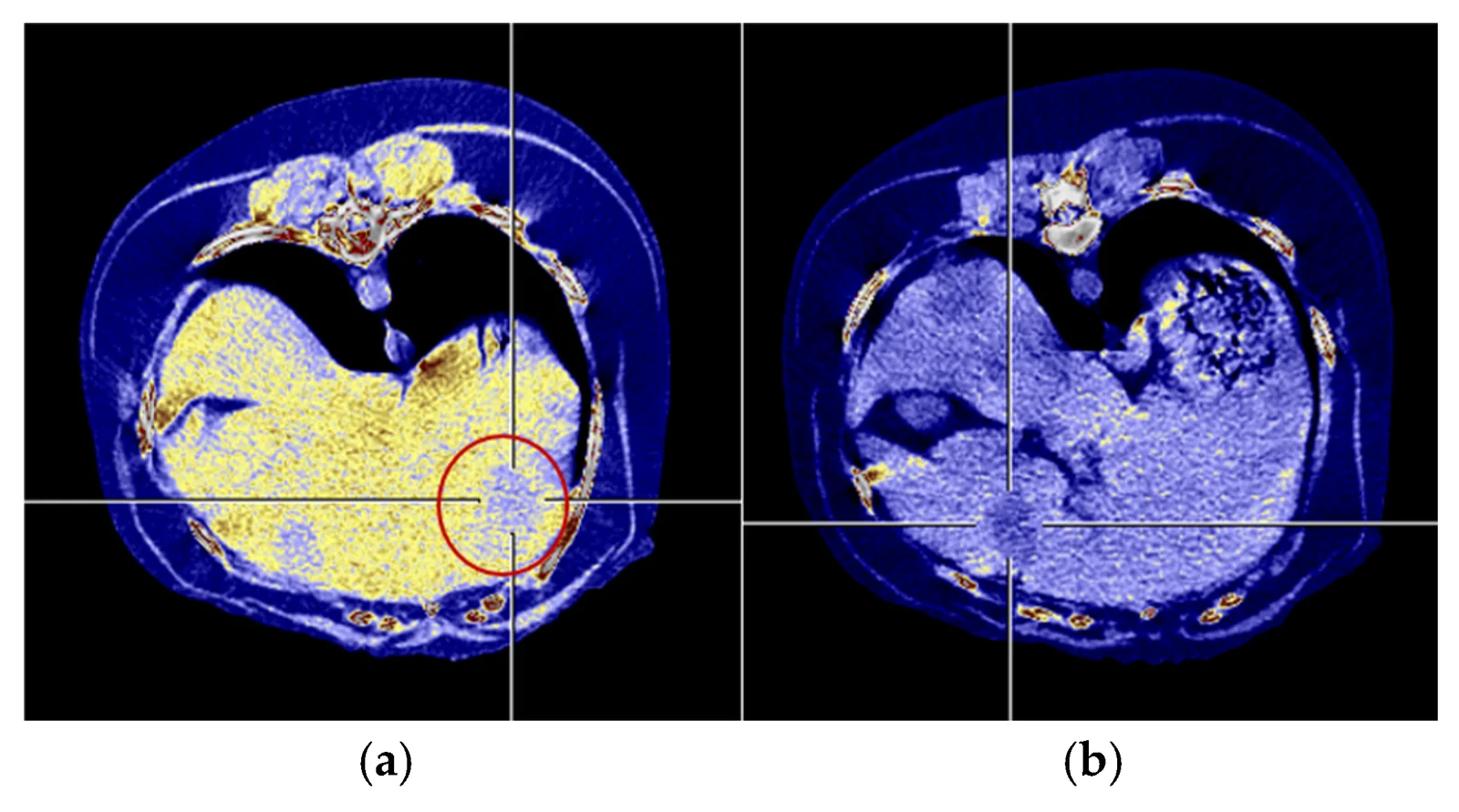
X-ray CT cross-sectional images of metastatic liver tumor lesions in the canine model acquired (**a**) 1 day and (**b**) 1 week after therapy, illustrating early post-treatment morphological changes.

X-ray CT-based segmentation and cross-sectional analysis demonstrated heterogeneous morphological responses of metastatic liver lesions following therapy. While some tumor foci exhibited a measurable reduction in volume, other lesions showed either limited growth or remained relatively stable in size, reflecting the intrinsically heterogeneous nature of metastatic tumor response. Nevertheless, when evaluated collectively, the overall tumor burden indicated a slight but consistent reduction. The phenomenon of heterogeneous partial tumor volume reduction is particularly illustrated in cross-sectional CT images of the liver (Figure 6), where two clearly distinguishable tumor lesions are visible one day post-administration, whereas only a single lesion remains detectable after one week. The disappearance of one lesion from the later image strongly suggests a localized therapeutic response, supporting the conclusion that the applied treatment induced a net antitumor effect despite inter-lesional variability.

## 3. Discussion

This study presents a complete and integrated workflow for ^103^Pd production, separation, purification, chelation, and in vivo evaluation, thereby establishing its feasibility as a theranostic Auger electron-emitting radionuclide. The upgraded RSE dry-distillation system demonstrated high separation efficiencies (64–86%) and recovery yields (81–94%), representing a substantial improvement over conventional wet-chemistry approaches. Traditional dissolution-based procedures are hindered by rhodium’s chemical inertness, lower overall yields, prolonged processing times, and the generation of considerable volumes of radioactive waste [19]. In contrast, the dry-distillation method, coupled with the novel substrate recovery process, offers a rapid, scalable, and waste-minimizing alternative suitable for repeated irradiations and high-throughput radionuclide production workflows.

In vivo SPECT/CT imaging demonstrated the successful application of NOTA- and DOTA-TATE-based ^103^Pd radioconjugates for preclinical imaging. The persistent tracer accumulation observed at multiple post-injection time points suggests sufficient in vivo stability for the performed imaging studies and supports the application of ^103^Pd complexes for preclinical imaging. When considered for particulate radioembolization of pulmonary lesions, ^103^Pd showed notable advantages over established β-emitters such as ^90^Y and ^177^Lu, which are known to exhibit deeper tissue penetration and can inadvertently irradiate adjacent healthy lung parenchyma [2,29]. Owing to its low-energy photon emissions and subcellular-range Auger electrons [30], ^103^Pd provides highly localized intratumoral energy deposition while minimizing off-target dose. This combination of imaging capability and enhanced microdosimetric selectivity highlights its promising theranostic profile. In this context, the use of serum albumin microspheres should be regarded as a physically retained particulate carrier rather than a molecular targeting agent, providing a biologically justified platform to explore localized pulmonary radioembolization concepts with ^103^Pd.

Preliminary translational evaluation in the canine model further supports the therapeutic potential of ^103^Pd. The slight reduction in tumor size, along with marked improvement in clinical condition, including restored mobility and an increase in glucose levels after previous hypoglycemia, suggests a biologically meaningful response. Although still exploratory, these early observations reinforce the rationale for continued investigation of ^103^Pd as a targeted therapeutic radionuclide.

A limitation of the present study is that dedicated in vitro stability studies and structural characterization of the Pd complexes were not performed. The primary objective of this work was to establish the cyclotron production, novel separation methodology, and proof-of-concept preclinical biological application of ^103^Pd rather than to perform a comparative evaluation of different Pd^2+^ chelator systems. Consequently, comprehensive stability investigations of the radioconjugates were beyond the scope of the present study. Nevertheless, high radiochemical purity, previous daughter-radionuclide release studies [16], and the in vivo biodistribution data obtained in this work suggested sufficient stability for the conducted imaging experiments. It should be noted, however, that Pd^2+^ exhibits coordination chemistry distinct from that of many routinely used radiometals and may present challenges for complexation with conventional macrocyclic chelators [31]. Recent studies have identified cyclam-based ligands as particularly promising chelator systems for palladium radionuclides [32,33]. Therefore, future work should include systematic serum stability investigations, structural characterization of the Pd complexes, and direct comparison of DOTA-, NOTA-, and cyclam-based chelators for ^103^Pd radiopharmaceutical applications.

In addition, future studies should focus on comprehensive dosimetric modeling, expanded therapeutic investigations, dedicated in vitro stability evaluation, and direct comparison with other Auger electron-emitting radionuclides to further elucidate the clinical translation potential of the ^103^Pd/^103m^Rh system.

## 4. Materials and Methods

Rhodium metal foils (99.85% purity; 125, 25, 12, and 6 µm; Goodfellow Cambridge Ltd., Huntingdon, UK) were used as cyclotron targets. DOWEX^®^ 1X8 anion-exchange resin (chloride form, 200–400 mesh; Sigma Aldrich–Merck, St. Louis, MO, USA) and analytical-grade reagents, including hydrochloric acid (HCl) (6 M), nitric acid (HNO_3_) (65% *w*/*w*), ammonium hydroxide (NH_4_OH) (28–30% *w*/*w*), ammonium acetate (CH_3_CO_2_NH_4_) (0.4 M, pH 5.5), methanol, acetone and physiological saline (0.9% NaCl), and gentisic acid, were obtained from Sigma Aldrich–Merck. NOTA (1,4,7-triazacyclononane-1,4,7-triacetic acid) and DOTA-TATE (DOTA-[Tyr^3^]-octreotate) and gentisic acid were purchased from ABX GmbH (Radeberg, Germany). NOTA-NHS ester (2,2-(7-(2-((2,5-dioxopyrrolidin-1-yl)oxy)-2-oxoethyl)-1,4,7-triazonane-1,4-diyl)diacetic acid) was purchased from CheMatech (Dijon, France), and ROTOP-HSA microspheres B20 were obtained from ROTOP Pharmaka GmbH (Dresden, Germany). All solutions were prepared using deionized water produced by a Milli-Q^®^ purification system (Merck Millipore, Darmstadt, Germany).

The yttria-stabilized zirconia (YSZ) crucibles were purchased from Goodfellow Cambridge Ltd., and the alumina ceramic crucible substrates were obtained from Kurt J. Lesker Company GmbH, Fritz-Schreiter-Strasse 18, 01259 Dresden, Germany. Low-binding microcentrifuge tubes (Eppendorf, Germany), iTLC-SG plates (Agilent Technologies, Santa Clara, CA, USA), C18 mini-column chromatographic separation (Sep-Pak^®^ C18, Waters Corporation, Milford, MA, USA), and Raytest Mini-GITA radio-TLC scanner (Raytest Isotopenmessgeräte GmbH, Straubenhardt, Germany) were used for radiochemical analyses. Irradiations were performed using an MGC-20 cyclotron (HUN-REN Institute for Nuclear Research (ATOMKI), Debrecen, Hungary). Activity measurements were carried out with an Atomlab™ 500 dose calibrator (Mirion Technologies, Atlanta, GA, USA), and gamma spectrometry was performed with a Canberra high-purity germanium (HPGe) detector (Model 2002 CSL; Canberra Industries, Meriden, CT, USA) and Genie-2000 (V3.4.1, 2016) software. Radiolabeling reactions were conducted in a Thermomixer^®^ C (Eppendorf SE, Hamburg, Germany). Centrifugation steps were performed using a benchtop centrifuge (Rotofix 32A, Hettich, Germany). NMRI nu/nu (nude) mice were obtained from Charles River Laboratories (Sulzfeld, Germany). SPECT/CT imaging and data processing employed a dedicated small animal high-resolution and high-sensitivity nanoScan^®^ SPECT/CT instrument and a clinical PET/SPECT/CT triple modality imaging system (Nucline™ acquisition software (version 3.04.014.0000) and InterView™ FUSION™ analysis software (version 1.2), all from Mediso Ltd., Budapest, Hungary).

### 4.1. Cyclotron Irradiation of ^103^Rh and Production of ^103^Pd

^103^Pd was produced via the ^103^Rh(p,n)^103^Pd nuclear reaction by irradiating rhodium metal foils with 11 MeV protons using an MGC-20 cyclotron at the HUN-REN Institute for Nuclear Research (ATOMKI, Debrecen, Hungary). The ^103^Rh(p,n)^103^Pd reaction is a well-established and widely documented cyclotron-based production route for ^103^Pd [34], offering favorable yields at proton energies within the 8–12 MeV range [17,35]. Rhodium foils with thicknesses of 6, 12, 25, and 125 μm were arranged in a stacked-foil configuration, from thickest to thinnest, on a copper backing plate serving as the cyclotron target holder. The assembled target stack was irradiated with a 20 μA proton beam for 30 h (h), yielding approximately 6 GBq of ^103^Pd at end-of-beam.

### 4.2. Improved Separation of ^103^Pd from Irradiated ^103^Rh

**Figure 7:**
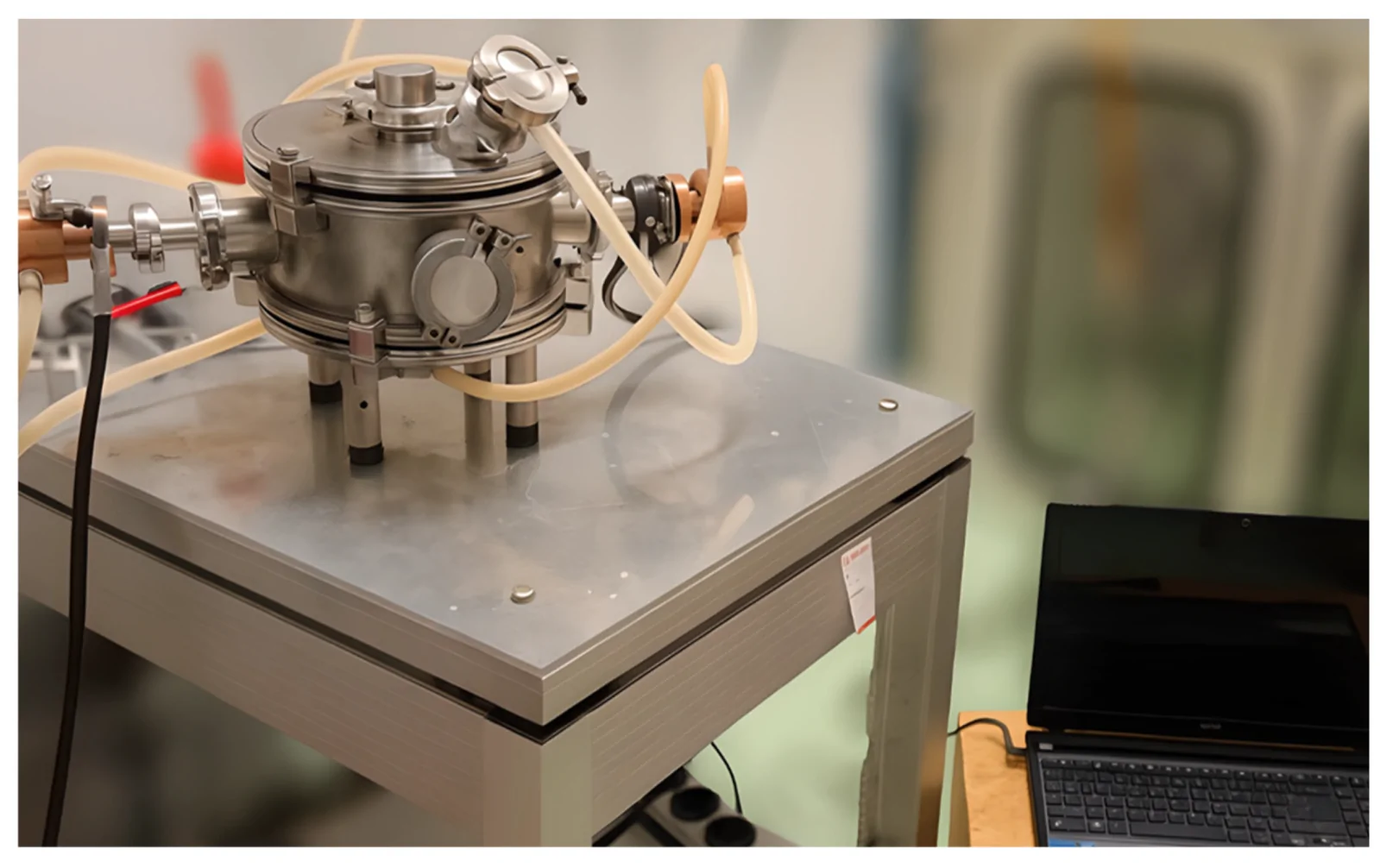
Upgraded, remotely controlled radionuclide separation equipment (RSE) incorporating improved components.

The separation of ^103^Pd from the irradiated rhodium targets was performed using custom-built radionuclide separation equipment (RSE) developed in our laboratory [20]. The system operates on a dry-distillation principle, in which ^103^Pd ions are selectively volatilized from the irradiated ^103^Rh foils and subsequently collected onto a dedicated deposition substrate. The RSE was recently upgraded by incorporating newly designed components to improve thermal stability, vacuum performance, and collection efficiency (Figure 7).

In the improved configuration, the previous collection substrate was replaced with an alumina ceramic crucible coated with a thin copper layer. This copper-coated alumina ceramic crucible substrate proved highly effective in promoting condensation of the volatilized ^103^Pd and substantially simplifying its subsequent recovery. For each separation experiment, three irradiated rhodium foils of different thicknesses were positioned vertically inside an yttria-stabilized zirconia (YSZ) crucible within the RSE.

The heating system was remotely controlled and powered by a TDK-Lambda Genesys 180A/8V unit, delivering a total heating power of ~550 W for a 24 h separation cycle. During operation, the crucible temperature was set at 1200 °C under high-vacuum conditions of 3–5 × 10^−5^ mbar, parameters optimized to maximize volatilization and efficient transfer of ^103^Pd-ions onto the copper-coated alumina ceramic crucible collection substrate.

### 4.3. Enhanced Recovery and Purification of ^103^Pd

**Figure 8:**
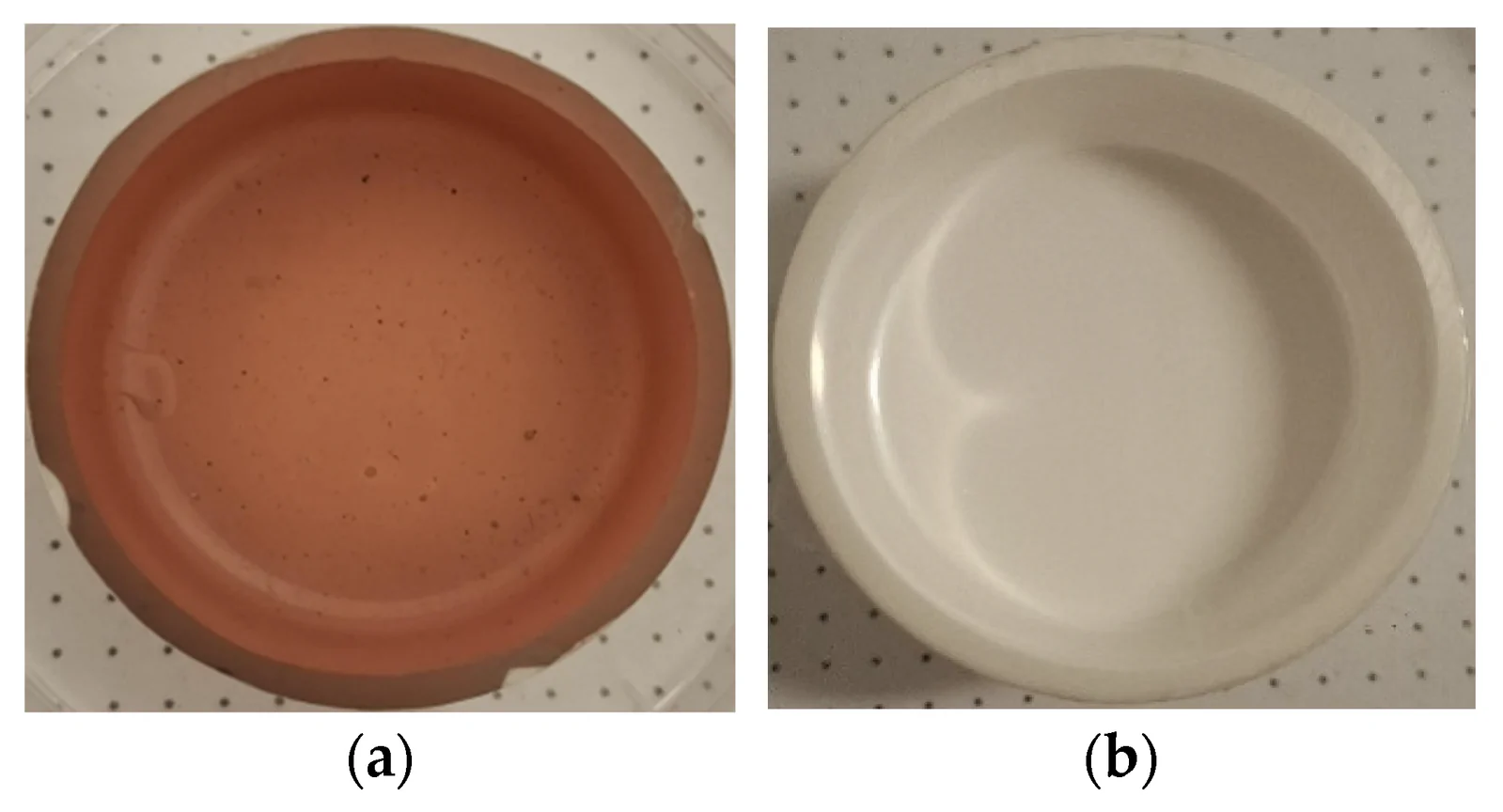
Alumina ceramic crucible substrate coated with a thin copper layer used for condensation of volatilized ^103^Pd: (**a**) before acidic treatment; (**b**) after acidic treatment.

The ^103^Pd activity collected on the copper-coated alumina ceramic crucible substrate was recovered by an optimized acidic extraction procedure. Several extraction media and approaches were evaluated, and the most effective method involved wiping the surface of the substrate with cotton moistened with 0.5–1 mL of nitric acid, followed by immersing the cotton in 2 mL of hydrochloric acid for >24 h. The cotton–acid mixture was then filtered, and the resulting eluate was passed through an anion-exchange resin for purification of ^103^Pd. After loading, the column was washed sequentially with hydrochloric acid and water to remove residual impurities, and purified ^103^Pd was subsequently eluted with 1 mL of ammonium hydroxide. This eluent choice was based on prior experimental optimization, in which ammonium hydroxide consistently provided the highest recovery and selectivity for ^103^Pd. The purified ^103^Pd solution was then heated to evaporate residual ammonia and reconstituted in 500 μL of 0.05 M HCl to obtain the final radiochemically pure ^103^Pd. After recovering the ^103^Pd activity from the alumina ceramic crucible substrate, the substrate retained its integrity and could be reused in subsequent separation runs after reapplying the copper coating (Figure 8).

### 4.4. Radiolabeling of [^103^Pd]Pd–NOTA and Functionalization of Biomolecular Conjugates

For the present study, a NOTA-NHS coupling approach was applied. The reaction was performed using a pH 8.4 sodium bicarbonate buffer, with a 20-fold molar excess of NOTA-NHS dissolved in DMSO, followed by 4 h incubation at room temperature in low-affinity reaction tubes under dark conditions. The reaction was quenched with 1 M Tris buffer, and the product was purified using a desalination column equilibrated with phosphate-buffered saline (PBS). Based on calculations corrected for molar mass and nitrogen content, approximately 3 × 10^−7^ mol chelator per milligram of microsphere (≈150,000 microspheres per mg) could be coupled, corresponding to approximately 1.8 × 10^12^ NOTA molecules per particle [36,37].

**Figure 9:**
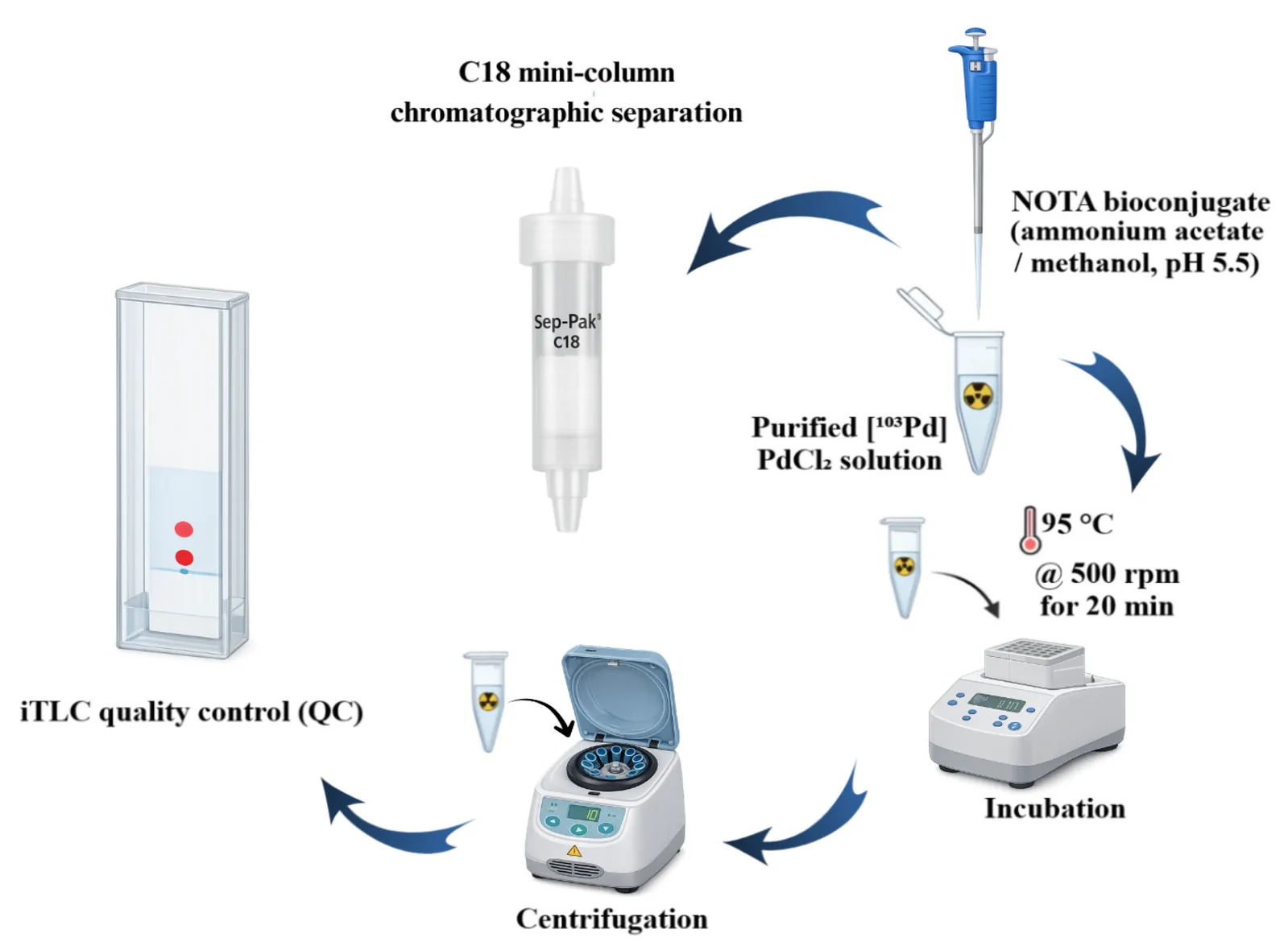
Radiolabeling protocol for the preparation of [^103^Pd]Pd–NOTA bioconjugates and radiochemical quality control.

For radiolabeling, the purified [^103^Pd]PdCl_2_ solution (≤100 μL per reaction) was mixed with the NOTA-bioconjugate, dissolved in 100 μL of 0.1 M acetate buffer at pH 5.5, resulting in a final reaction volume of 200 μL. The mixture was incubated in a thermomixer (500 rpm) at 95 °C for 20 min to facilitate chelation, after which the mixture was briefly centrifuged and allowed to cool to room temperature. After cooling to room temperature, radiochemical yield was assessed by C18 mini-column chromatographic separation and instant thin-layer chromatography (iTLC) using 1 M ammonium acetate and methanol as the mobile phase (Figure 9). Prior to administration, the radiolabeled solution was diluted with physiological saline to the required volume and activity concentration.

Using this approach, serum albumin microspheres were successfully functionalized with NOTA and radiolabeled with ^103^Pd for lung-targeted deposition studies.

### 4.5. Radiolabeling of [^103^Pd]Pd–DOTA-TATE

Radiolabeling of the purified ^103^Pd with the DOTA-TATE (DOTA-[Tyr^3^]-octreotate) peptide was performed using a standardized metal-ion complexation protocol adapted from established procedures [38]. The DOTA-TATE peptide was supplied in lyophilized form and reconstituted in sodium acetate buffer prior to use.

For radiolabeling, 4 mg of gentisic acid (radioprotectant) was dissolved in 0.8 mL of 0.4 M sodium acetate (NaOAc) buffer. Subsequently, a palladium-103 chloride solution containing 712 MBq of activity in 0.68 mL was added to the buffered gentisic acid solution. Thereafter, 40 μg of DOTA-TATE peptide was introduced into the reaction mixture. The resulting solution was incubated at 95 °C for 25 min to promote complex formation. After incubation, the vial was briefly centrifuged (10 s) and allowed to cool to room temperature prior to quality control analysis.

### 4.6. Administration and Imaging Protocol of [^103^Pd]Pd–NOTA in Murine Models

NMRI Nu/Nu mice were used for the imaging studies. Animals received an intravenous injection of 100 μL of [^103^Pd]Pd–NOTA, corresponding to cca. 60 MBq per mouse. SPECT/CT acquisitions were performed at multiple time points following tracer administration. All imaging procedures were conducted under isoflurane anesthesia, which was maintained using 1.5–2% isoflurane in medical oxygen, adjusted according to the animal’s respiration rate.

**Figure 10:**
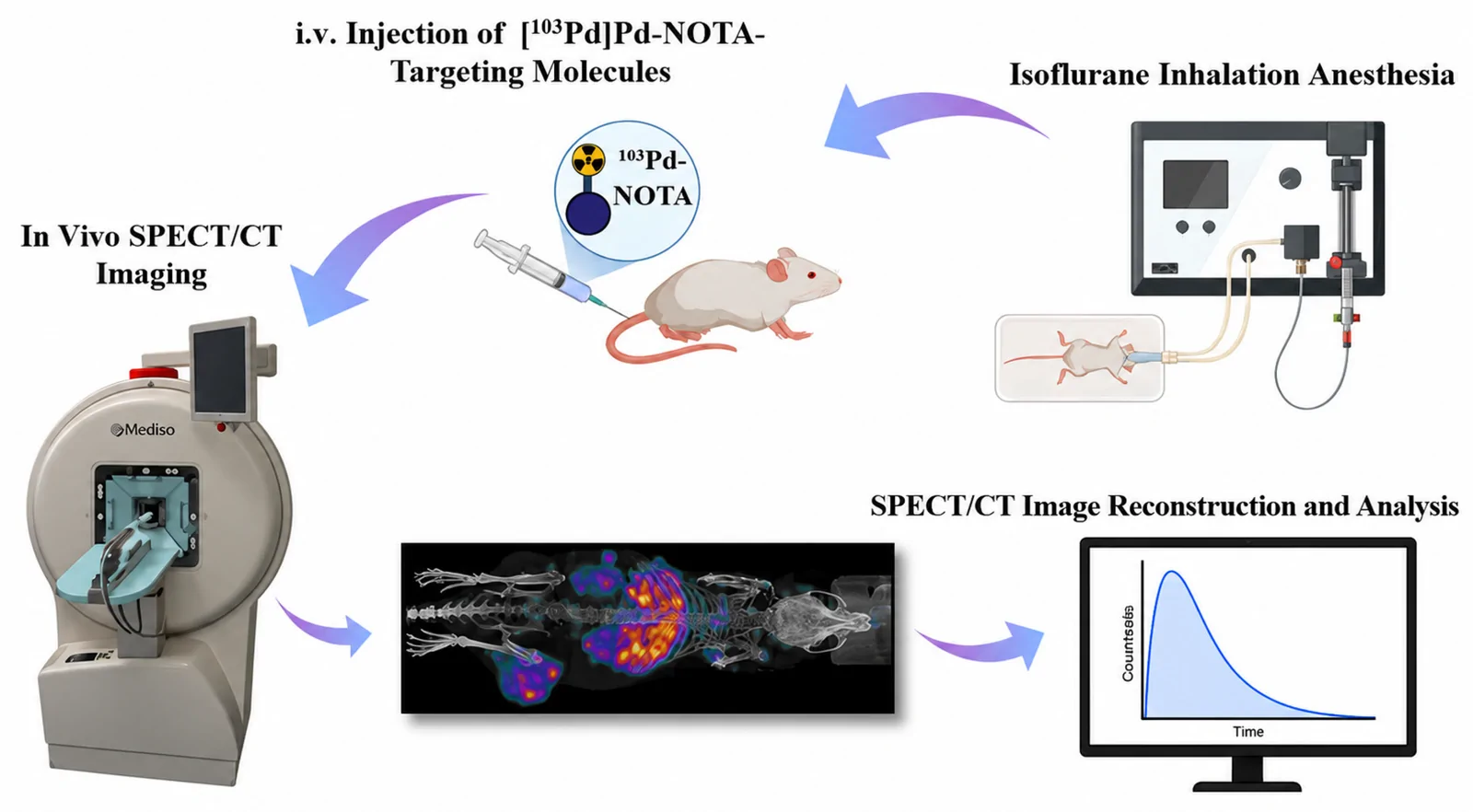
Experimental workflow for [^103^Pd]Pd–NOTA administration and nanoSPECT/CT imaging in murine models.

Imaging was performed using a dedicated small animal high-resolution and high-sensitivity nanoScan^®^ SPECT/CT instrument (Mediso Ltd., Budapest, Hungary) equipped with multi-pinhole collimators (Figure 10). For ^103^Pd, a high-energy tungsten collimator (aperture 65), recommended by the manufacturer for isotopes with gamma emissions above 250 keV, was applied.

Gamma-ray emissions and their relative abundances were processed using the Nucline acquisition software (Mediso Ltd., Budapest, Hungary) as summarized in Table 3. Of the three detectable emissions of ^103^Pd, only the primary 20.5 keV peak was used for SPECT image acquisition, applying a 20% energy window centered on this peak. However, due to the presence of high-energy peaks, high stopping-power collimators were required to suppress beam-related artifacts.

Whole-body SPECT/CT imaging was performed using 30 frames per cycle with a termination condition of 120 s per frame, covering a longitudinal scan range of 128 mm. SPECT data were reconstructed using 0.2 mm isovoxels. Co-registered X-ray CT images were acquired using a 55 kV source voltage in a cone-beam spiral mode system with a pitch of 2.5. Semiquantitative analysis of SPECT data was performed by measuring counts per unit volume (counts/mL). Image processing and analysis were performed using the Fusion software package (Mediso Ltd., Budapest, Hungary).

### 4.7. Administration and Imaging Protocol of [^103^Pd]Pd–DOTA-TATE in a Canine Model of Spontaneous Liver Cancer

**Figure 11:**
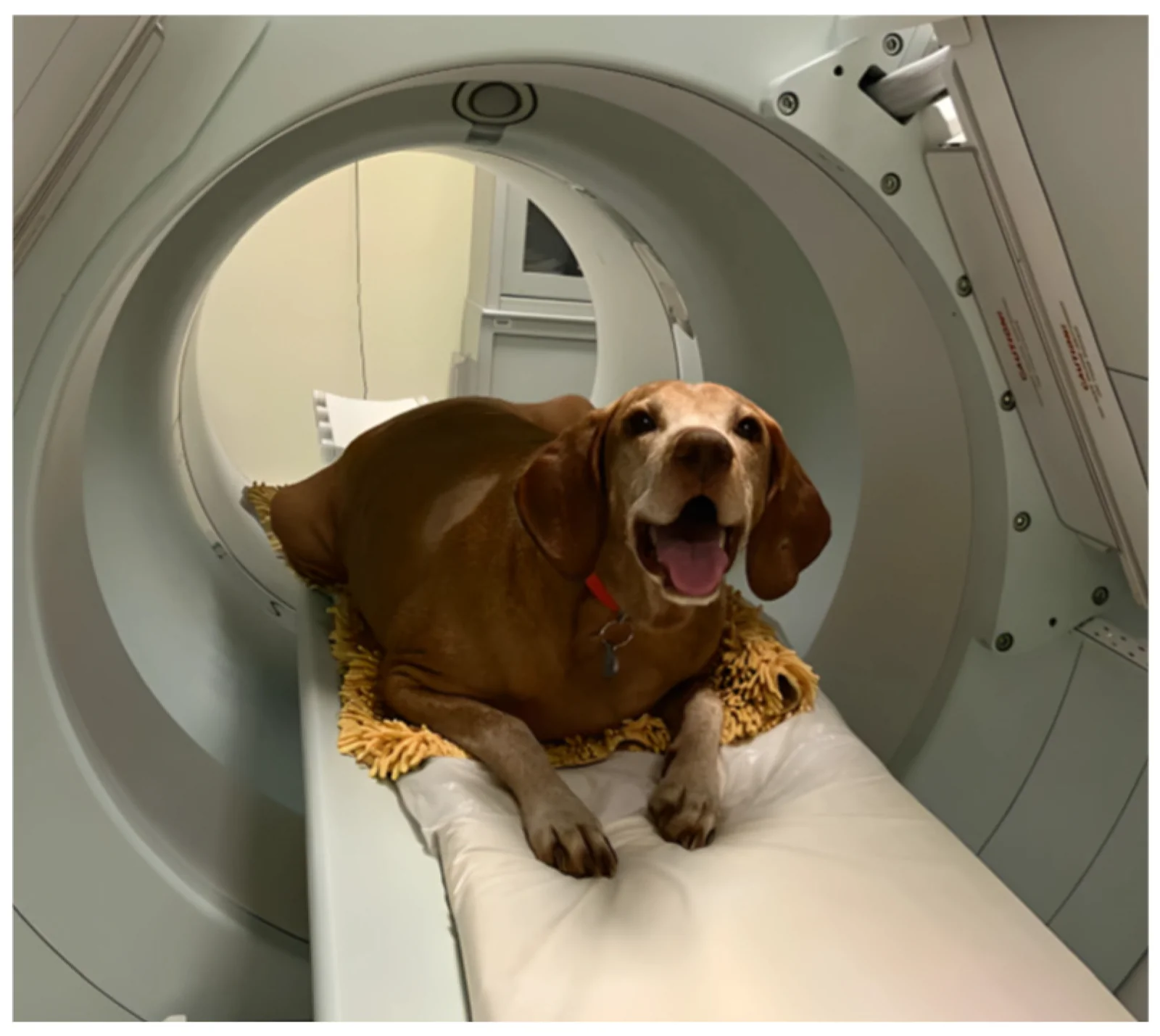
Canine model during SPECT/CT imaging with [^103^Pd]Pd–DOTA-TATE.

Use of a naturally occurring canine malignancy provides a clinically relevant intermediate model bridging rodent studies and potential human applications. All procedures were conducted with the owner’s consent and in accordance with institutional veterinary ethical guidelines. A 12.5-year-old female Hungarian Vizsla family dog diagnosed with a histologically confirmed, spontaneous, and metastatic late-stage insulinoma exhibiting somatostatin receptor subtype-2 (SSTR2) expression was included as a translational large-animal model for imaging and therapeutic investigation (Figure 11). The dog received a single intravenous administration of approximately 520 MBq of [^103^Pd]Pd–DOTA-TATE solution diluted with physiological saline in a total injection volume of 2 mL. Thereafter, continuous veterinary monitoring was performed, including regular assessment of blood glucose levels to detect episodes of hypo- or hyperglycaemia. A trial acquisition of SPECT/CT imaging data was subsequently performed at 1 h post-injection to monitor tracer biodistribution and tumor uptake over time. Notably, no anesthesia was required during image acquisition, as the animal remained fully calm and cooperative throughout the scanning procedures, but this clinically important feature decreased the available time window for imaging to less than 60 min.

X-ray Computed Tomography imaging to monitor lesion size in the liver of the dog was conducted at Semmelweis University Clinic of Medical Imaging (Budapest, Hungary) using the CT component of a PET/SPECT/CT trimodality system (AnyScan TRIO, Mediso Ltd., Budapest, Hungary). Only CT anatomical imaging was feasible in the large tissue volume and clinically available very short imaging time window for the spontaneously diseased dog. CT images were then processed using the dedicated Fusion (Mediso Ltd., Budapest, Hungary) software using automatic Hounsfield-unit-based segmentation.

All animal experiments were conducted in accordance with the ARRIVE guidelines and the 3R principles. The studies were approved by the local and national animal welfare authority, the Pest County Government Office, under registration number PE/EA/00929-5/2021 for the rodent experiments and for the dog studies, which latter also carried the full consensual signature of the owner of the dog.

## 5. Conclusions

In summary, our work outlines a practical production-to-in vivo pipeline for ^103^Pd, enabling its application as a highly localized Auger-based radionuclide. The upgraded dry-distillation system achieved high separation and recovery efficiencies, while in vivo SPECT/CT imaging confirmed the biological suitability of [^103^Pd]Pd–NOTA and [^103^Pd]Pd–DOTA-TATE complexes. The incorporation of ^103^Pd into an HSA-based particulate system further suggests a feasible extension of established brachytherapy principles toward microscale radioembolic applications. Preliminary murine and canine data indicate localized tumor uptake and early therapeutic effects, supporting further development of ^103^Pd for targeted radiotherapy applications.

## Figures and Tables

**Table 1:** Principal photon emissions associated with the decay of ^103^Pd and ^103m^Rh [15].

Radionuclide	Radiation	Energy (keV)	Intensity (%)
^103^Pd	X-ray	2.376–3.408	8.73
^103^Pd	X-ray	20.073	22.09
^103^Pd	X-ray	20.215	41.83
^103^Pd	X-ray	22.699–22.912	11.36
^103^Pd	X-ray	22.699–23.215	13.25
^103^Pd	X-ray	23.167–23.172	1.89
^103^Pd	γ-ray	39.749	0.0683
^103^Pd	γ-ray	53.297	0.00003
^103^Pd	γ-ray	62.413	0.00104
^103^Pd	γ-ray	241.885	0.0000005
^103^Pd	γ-ray	294.988	0.00280
^103^Pd	γ-ray	317.725	0.000015
^103^Pd	γ-ray	357.458	0.0221
^103^Pd	γ-ray	443.795	0.000015
^103^Pd	γ-ray	497.080	0.00396
^103m^Rh	X-ray	2.376–3.408	3.93
^103m^Rh	X-ray	20.073	2.01
^103m^Rh	X-ray	20.215	3.84
^103m^Rh	X-ray	22.699–22.912	1.03
^103m^Rh	X-ray	22.699–23.215	1.21
^103m^Rh	X-ray	23.167–23.172	0.172
^103m^Rh	γ-ray	39.755	0.0685

**Table 2:** Summary of ^103^Pd evaporation yields under different experimental conditions.

	Substrate	T_evap_	∆t_evap_	Foil Thickness	Evaporation Yield (%)			
		°C	h	µm	Run 1	Run 2	Run 3	Run 4
Improved separation experiments	Alumina	1200	24	12/12/6	74	83	64	86

**Table 3:** Detectable gamma-ray and X-ray emissions of ^103^Pd and their corresponding intensities for SPECT imaging.

Name	Peak (KeV)	Intensity (%)
Primary	20.50	76.930
Secondary	39.75	0.068
Tertiary	357.45	0.022

## Data Availability

Data are contained within the article.

## Abbreviations

The following abbreviations are used in this manuscript:

DMSO	Dimethyl sulfoxide
DOTA-TATE	DOTA-[Tyr^3^]-octreotate
HPGe	High-Purity Germanium detector
HSA	Human Serum Albumin
iTLC	Instant Thin-Layer Chromatography
NOTA	1,4,7-Triazacyclononane-1,4,7-triacetic acid
NHS	N-Hydroxysuccinimide
PBS	Phosphate-Buffered Saline
PRRT	Peptide Receptor Radionuclide Therapy
RSE	Radionuclide Separation Equipment
RCP	Radiochemical Purity
SSTR2	Somatostatin Receptor Subtype 2
YSZ	Yttria-Stabilized Zirconia
